# An Unusual Presentation of T-cell/Histiocyte-Rich B-cell Lymphoma: A Diagnostic Challenge

**DOI:** 10.7759/cureus.93191

**Published:** 2025-09-25

**Authors:** Joshita Reddy, Steve Thomas, Barathi Gunabooshanam

**Affiliations:** 1 Department of General Medicine, Sri Ramachandra Institute of Higher Education and Research, Chennai, IND; 2 Department of Haematology, Sri Ramachandra Institute of Higher Education and Research, Chennai, IND; 3 Department of Pathology, Sri Ramachandra Institute of Higher Education and Research, Chennai, IND

**Keywords:** aggressive b-cell lymphoma, extranodal presentation, immunohistochemistry in lymphoma, necrotizing lesion, t-cell/histiocyte rich large b-cell lymphoma

## Abstract

Necrotizing masses pose a remarkable diagnostic challenge as they masquerade as infections, ischemic events, malignancies, autoimmune conditions, etc. Herewith, we are discussing a rare presentation of a man in his early 40s who presented with a necrotizing mass in the anterior chest wall and underwent multiple evaluations resulting in several initial misdiagnoses before the definitive diagnosis of T-cell/histiocyte-rich B-cell lymphoma was made. The patient was initiated on the R-CHOP (rituximab, cyclophosphamide, Doxorubicin, Vincristine, and prednisone) regimen and is currently under therapy. This case report highlights the significance of the diagnostic approach with thorough histopathological and immunohistochemical evaluation to diagnose unusual presentations, for appropriate management, and improving patient outcomes.

## Introduction

T-cell/histiocyte-rich large B-cell lymphoma (THRLBCL) is an uncommon, aggressive morphological variant of diffuse large B-cell lymphoma (DLBCL), comprising only 1-3% of all cases [1-3]. Owing to its rarity and distinctive histological features, it is recognized as a separate entity in the World Health Organization (WHO) classification of lymphoid neoplasms [4]. Clinically, THRLBCL most often presents in middle-aged individuals with a notable male predominance and typically manifests at advanced clinical stages (III/IV) [3,5].

Patients usually present with generalized lymphadenopathy accompanied by frequent extranodal involvement. The spleen, liver, and bone marrow are the most commonly affected sites, though unusual extranodal presentations, including mediastinal and chest wall disease, have been reported [3,5]. In addition, systemic “B symptoms” such as fever, night sweats, and unintentional weight loss are common, reflecting the aggressive clinical behavior of the disease [5]. Other conditions like tuberculosis, fungal infections, autoimmune causes, etc., can present with similar clinical features.

From a pathological standpoint, THRLBCL is characterized by a striking histological pattern in which neoplastic large B cells are few in number and scattered singly within a background dominated by abundant reactive T lymphocytes and histiocytes. This unique microenvironment often masks the malignant component and closely mimics benign reactive or inflammatory conditions. Consequently, patients are at high risk of misdiagnosis, with cases frequently mistaken for reactive lymphoid hyperplasia, T-cell-rich disorders, or even Hodgkin lymphoma, particularly nodular lymphocyte-predominant Hodgkin lymphoma [5]. Hence, immunohistochemistry (IHC) is indispensable in establishing the diagnosis due to the unique nature of THRLBCL. [4,5].

Because of these diagnostic complexities, THRLBCL represents a clinical and pathological challenge. Failure to recognize the entity early can result in delayed treatment initiation and poorer clinical outcomes. This case report describes a rare presentation of THRLBCL as a necrotizing mass in the anterior chest wall, highlighting the diagnostic pitfalls, the essential role of histopathological and immunohistochemical evaluation, and the importance of timely recognition for appropriate management and improved patient outcomes [1,3].

## Case presentation

A previously healthy middle-aged man in his early 40s with no prior comorbidities presented with complaints of painless right axillary swelling, which progressively extended into the right anterior chest wall over the past seven months, with ulceration associated with intermittent episodes of high-grade fever. He also complained of unintentional weight loss and a significant reduction in appetite. He had no prior history of tuberculosis, chronic illnesses, or trauma to the affected area.

**Figure 1:**
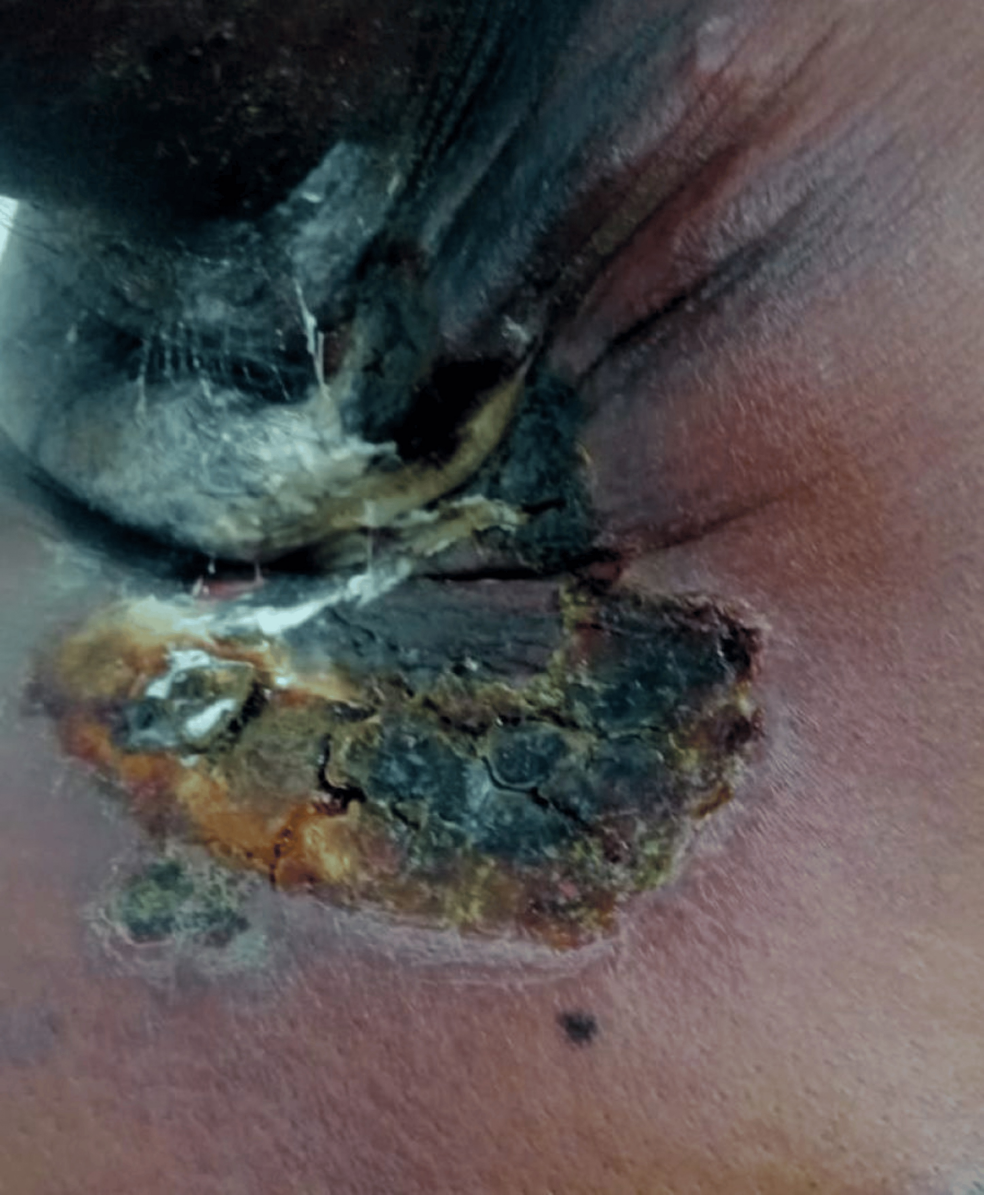
Right axillary and right anterior chest wall mass with overlying skin ulceration measuring 6 × 4 cm and purulent discharge.

Initial evaluation at an outside hospital included empirical treatment with antibiotics, followed by a fine-needle aspiration cytology (FNAC), which revealed spindle cells and atypical cells. A subsequent core biopsy demonstrated granulomatous inflammation. In light of a positive Mantoux test and histopathological findings, the patient was diagnosed with presumed tuberculous lymphadenitis and started on anti-tuberculous therapy (ATT). Despite 5 months of ATT, the patient showed no clinical improvement, prompting referral to our tertiary care center. On physical examination, a firm, non-tender, irregular swelling measuring approximately 20 × 15 × 10 cm was observed over the right anterior and lateral chest wall, extending up to the level of the nipple. The mass had overlying skin ulceration measuring 6 × 4 cm with purulent discharge (Figure 1). No presence of generalized lymphadenopathy. Abdominal examination revealed no hepatosplenomegaly. Other systems examination was found to be unremarkable.

Investigations

**Figure 2:**
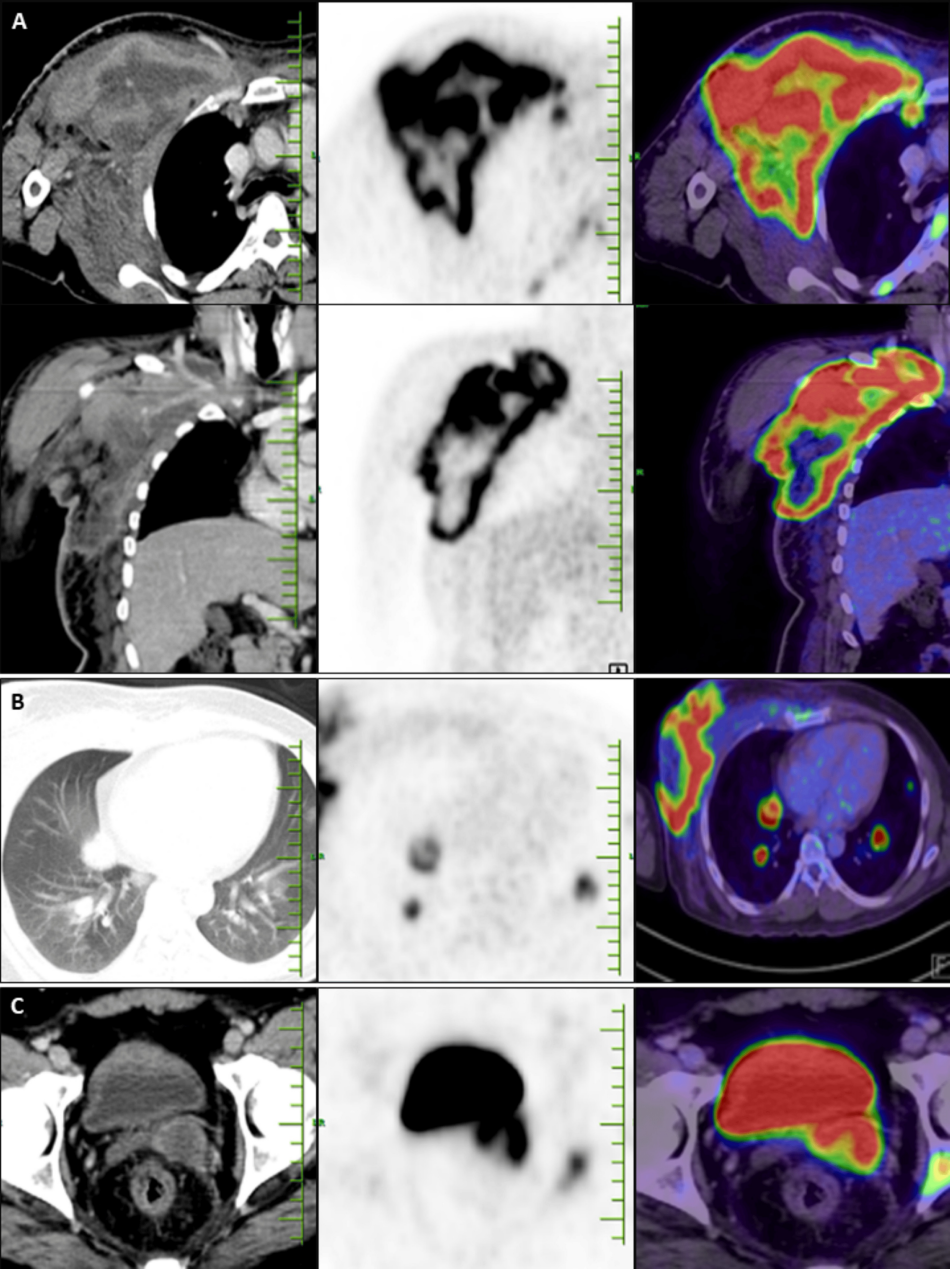
PET-CT Images Before Starting Chemotherapy. A large, ill-defined, geographically shaped FDG avid (SUV max 18.8) necrotic mass lesion was seen involving the muscular plane of the right anterolateral chest wall, measuring ~14.0 × 17.5 × 19.5 cm (AP × TR × CC); (B) Multiple FDG avid (SUV max 7.5) nodules seen in bilateral lungs, the largest measuring 29 × 22 mm in the right lower lobe; (C) FDG avid (SUV max 13.4) hypoenhancing lesion seen in the left seminal vesicle, measuring 32 × 23 mm-Metastatic deposits. FDG: fluorodeoxyglucose

Routine blood count and peripheral smear revealed leucopenia with thrombocytopenia (Table 1). Retroviral serology was found to be negative. Serum lactate dehydrogenase (LDH) was found to be 497 (elevated). The patient was initiated on empirical antibiotics, despite which the fever persisted. Given bicytopenia and suspicion of malignancy, bone marrow aspirate showed normocellular marrow with normal trilineage hematopoiesis. A bone marrow biopsy showed granulomatous inflammation. Histopathology of the axillary lymphoid block from an outside hospital showed features suggestive of granulomatous inflammation. Special stains for acid-fast bacilli were found to be negative. The differential diagnosis to be considered when a patient presents with a necrotizing mass includes infections, inflammations, malignancies, and traumatic or toxin-induced causes. Traumatic and toxin-induced etiologies were ruled out in this patient, as history and physical examination were not suggestive. Inflammatory causes are unlikely in this patient due to the absence of other systemic manifestations. As the above investigations were found to be inconclusive, with a strong suspicion of malignancy, a whole body PET-CT scan was done, which showed a large, ill-defined, geographic-shaped, necrotic, malignant-looking mass lesion in the right anterolateral chest wall, with metastasis to bilateral lungs, multiple visualized bones, and the left seminal vesicle (Figure 2). This raised suspicion for various malignancies like primary soft tissue sarcomas, osteosarcomas, metastatic carcinoma, lymphomas, etc. [6,7]

**Table 1:** Bicytopenia seen on serial monitoring.

CBC	Day 1	Day 4	Day 8
Hemoglobin (g/deciliter)	13.3	12.7	12.8
Total WBC (/millimeter cube)	3340	2630	2700
Neutrophils (Percentage)	66.4	54.3	69.3
Lymphocytes (Percentage)	24	31.6	18.1
Monocytes (Percentage)	9	11.8	10.4
Eosinophils (Percentage)	0	1.5	1.2
Basophils (Percentage)	0.3	0.4	0.3
Platelet count (Lakhs/microliter)	0.7	0.49	0.65

**Figure 3:**
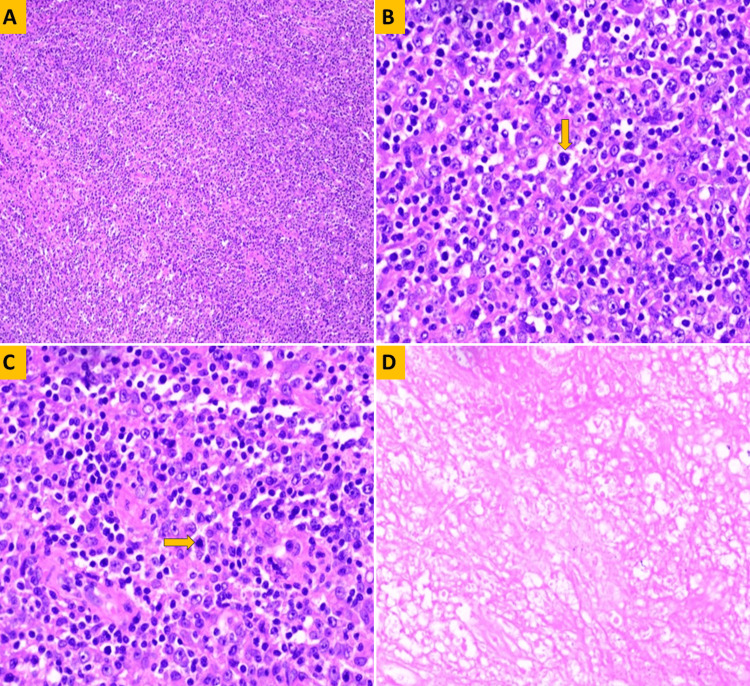
Histopathology Images Of Tissue From the Right Anterior Chest Wall. (A) Hematoxylin and eosin-stained sections from the soft tissue of the chest wall showing a diffuse population of lymphoid cells X100. (B) High power view of the section showing scattered neoplastic large B cells (yellow arrow pointer) morphologically resembling centroblasts are seen in a background of a reactive population of T cells and numerous histiocytes X400. (C) Frequent mitosis was noted ×400 (yellow arrow pointer). (D) Patchy areas of necrosis were also noted in the specimen examined.

**Figure 4:**
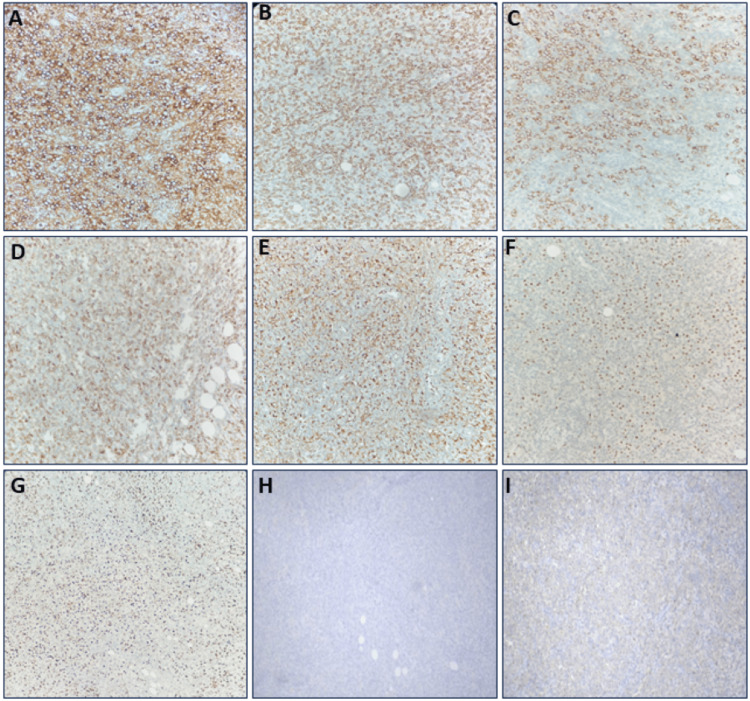
Immunohistochemistry Images. IHC shows diffuse positivity for (A) CD45, (B) positive for CD3, suggestive of reactive T cells,(C) positive for CD20 in the large B cells, (D) positive for CD5, suggestive of reactive T cells, (E) CD68 in the background histiocytes, (F) BCL6 scattered positivity. (G) Ki 67 is 60%.(H) IHC is Negative for CD15, (I) IHC is negative for CD30.

Tissue diagnosis is key to differentiating between infectious and malignant etiologies. Right supraclavicular node excision was done. Histopathology showed a polymorphous population of lymphocytes admixed with numerous histiocytes forming ill-defined granulomas. Tissue from the anterior chest wall showed predominantly necrosis with partial infiltration of viable neoplastic cells (Figure 3). Immunohistochemistry performed showed cells to be positive for CD45, CD20,CD3, CD5, CD68, BCL6 and negative for CD30, cyclin D1, MUM-1, CD1 and Epstein-Barr virus with a Ki67-60% (Figure 4). Evaluation for EBV was found to be negative. Together, these findings supported the diagnosis of THRLBCL.

**Figure 5:**
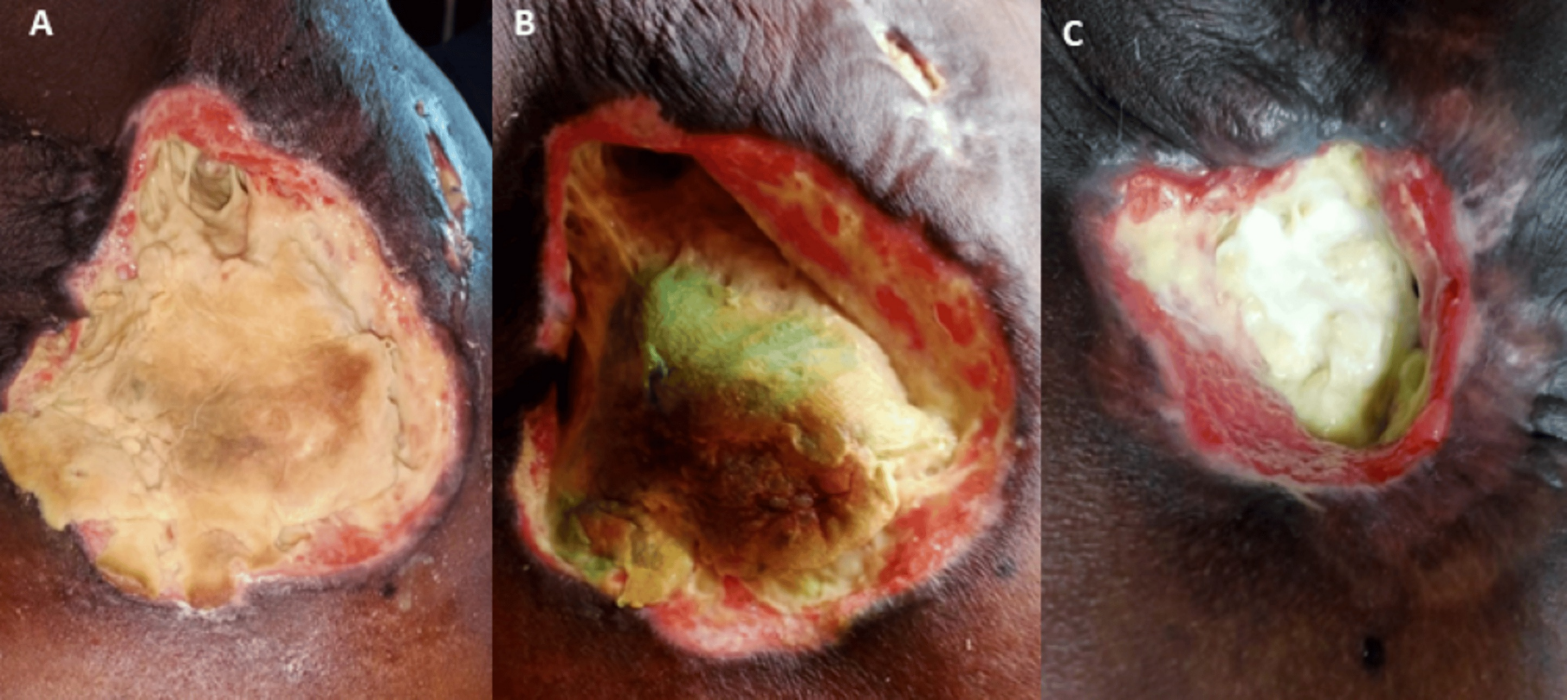
Images of Mass Post Chemotherapy Initiation. (A, B, C) Serial images showing a gradual reduction in the size of the tumor post initiation of chemotherapy.

**Figure 6:**
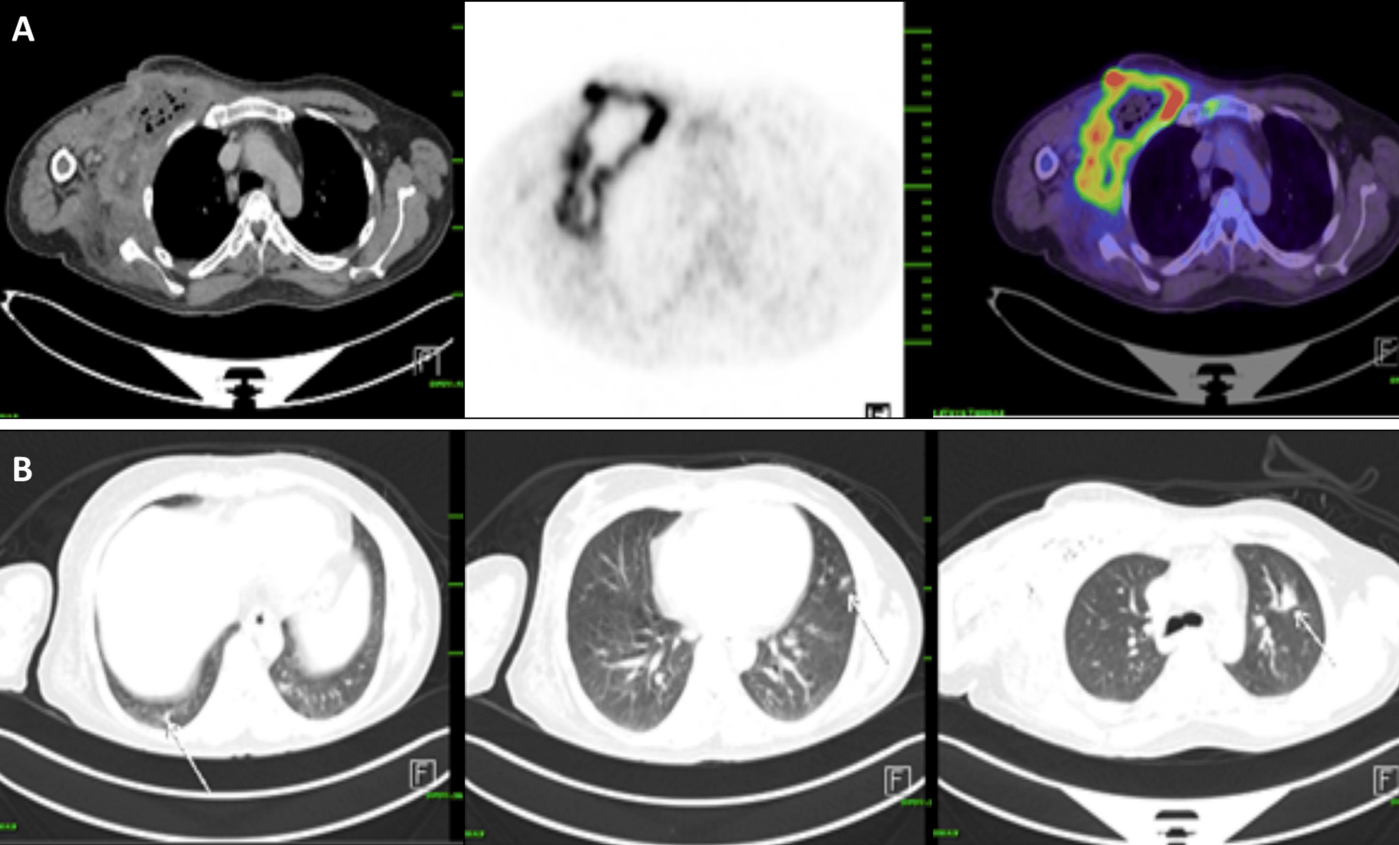
PET-CT Images Post Three Cycles of R-Chop Regimen. (A) A large ill marginated peripherally enhancing, peripherally FDG avid (SUV max 13, previously 20.0) necrotic mass lesion with multiple air foci within measuring ~ 5.6 × 10.5 cm (previously ~ 14 × 17.5) is seen involving the intermuscular plane of right anterolateral chest wall, seen extending up to skin – significant decrease in the size and metabolic activity is noted. (B)Significant reduction in the size with complete resolution of metabolic activity of the multiple FDG-avid nodules in bilateral lungs, measuring ~ 7 × 7 mm (previously 29 × 22 mm) in the posterior segment of the right lower lobe.

**Figure 7:**
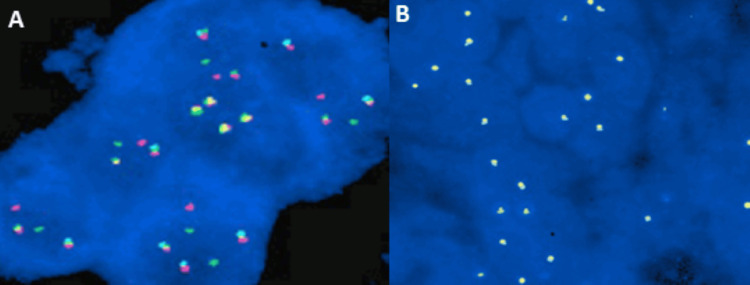
: Fluorescence In-Situ Hybridization (FISH) (A) BCL6 positivity of 24% present. (B) MYC negative

The patient was initiated on the R-CHOP (rituximab, cyclophosphamide, Doxorubicin, Vincristine, and prednisone) regimen [8-10]. Over the course of therapy, the patient developed febrile neutropenia, which was managed with appropriate supportive care and empirical antibiotics. The patient required recurrent wound debridement and management with appropriate antibiotics (Figure 5). A repeat PET-CT showed significant interval size reduction with a partial metabolic response in the right anterior chest wall lesion, significant size reduction and a complete resolution of metabolic activity in nodules of bilateral lungs, and complete resolution of size and metabolic activity in the lesion of the left seminal vesicle (Figure 6) [6,7]. The patient is currently undergoing chemotherapy and is under regular follow-up. During the course of therapy, fish analysis was done and was found to be positive for BCL6 (24%) (Figure 7).

## Discussion

Literature on THRLBCL is scarce owing to its rarity and the fact that it can be underdiagnosed as infective (TB, atypical mycobacterium, fungal), reactive immunoblastic proliferations, or misdiagnosed due to its close resemblance to nodular lymphocyte-predominant Hodgkin lymphoma (NLHPL) or classical Hodgkin lymphoma (CHL) [1-3,5]. The present case exemplifies these challenges.

Necrotizing granulomas were repeatedly observed in both lymph node and marrow samples, strongly suggesting tuberculosis in the context of a positive Mantoux test. However, the absence of clinical improvement on prolonged ATT, negative special stains for acid-fast bacilli, and negative fungal cultures necessitated further exploration. This highlights how necrotizing soft tissue granulomas, although commonly associated with infections, may occasionally be an atypical manifestation of an underlying lymphoma(usually presenting with “B symptoms”, peripheral lymphadenopathy, extranodal manifestations in liver, spleen, or bone marrow), requiring thorough evaluation.

Another diagnostic challenge is the overlap of histopathological features with Hodgkin lymphoma. Nodular lymphocyte-predominant Hodgkin lymphoma (NLPHL) and CHL may both present with lymph node enlargement, B symptoms, and scattered atypical cells in a reactive background. THRLBCL, however, differs in that the malignant B cells are few and dispersed, often mimicking reactive changes. In our case, immunohistochemistry was crucial: CD20 and BCL6 confirmed the B-cell lineage, CD3 and CD68 highlighted the abundant T cells and histiocytes typical of the microenvironment, and the absence of CD30/CD15 effectively ruled out CHL. This extended IHC panel was therefore indispensable in resolving the diagnostic ambiguity (Table 2).

The discordant bone marrow findings also posed a challenge. While aspirate showed normocellular marrow with intact hematopoiesis, the biopsy revealed granulomatous inflammation. This pattern of preserved hematopoiesis with nonspecific granulomas highlights the need for integrated clinical, imaging, and pathology data to distinguish from infective or malignant etiology.

**Table 2:** Key differences between CHL, NLPHL, THRLBCL + : positive; - : negative CHL: Classical Hodgkin lymphoma; NLPHL: nodular lymphocyte-predominant Hodgkin lymphoma; THRLBCL: T-cell/histiocyte-rich large B-cell lymphoma

Feature	Classical Hodgkin Lymphoma (CHL)	Nodular Lymphocyte-Predominant Hodgkin Lymphoma (NLPHL)	T-cell/Histiocyte-Rich Large B-cell Lymphoma (THRLBCL)	
				Epidemiology	Bimodal age (young adults and after 55 years), slight male predominance	Young to middle-aged men	Rare (1–3% of DLBCL), middle-aged, strong male predominance		
Typical Presentation	Cervical or mediastinal lymphadenopathy, B symptoms are common	Localized (peripheral nodes), usually indolent, B symptoms uncommon	Advanced stage (III/IV), generalized lymphadenopathy, frequent extranodal sites, B symptoms common						
				Histology	Reed-Sternberg cells in a mixed inflammatory background	“Popcorn” (Lymphocyte-predominant) cells in a nodular background of small B cells	Scattered malignant large B cells amid abundant reactive T cells and histiocytes		
Key Immunophenotype	CD30+, CD15+ (majority), PAX5 weak+, CD20–/weak, CD45–	CD20+, CD45+, BCL6+, EMA+, usually CD30–, CD15–	CD20+, CD45+, BCL6+, CD3+, CD68+, variable expression of MUM1, CD30–, CD15–						

Molecular studies have further refined the understanding of the pathogenesis of THRLBCL. The unique cellular milieu suggests a critical role of the tumor microenvironment in the pathogenesis and progression of the disease. This environment reflects immune evasion and immune modulation mechanisms by the tumor cells. The pathogenesis of THRLBCL involves several genetic alterations also seen in DLBCL, including overexpression of BCL2 and mutations in TNFAIP3 and MYD88 (L265P), which promote tumor cell survival. Epigenetic regulators such as CREBBP and EP300 are frequently mutated, disrupting transcriptional control. Recurrent chromosomal gains (3q, 18q) and losses (6q) further characterize the disease [2,8,9]. Importantly, amplification of 9p24.1 leads to PD-L1/PD-L2 overexpression, contributing to immune evasion [8]. These insights underscore the therapeutic potential of immune checkpoint inhibitors, particularly in refractory THRLBCL with extensive T-cell infiltration.

As THRLBCL is an aggressive disease, usually presenting as an advanced disease, therapy must be intensive and prompt. R-CHOP regimen is the first line of management, administered every 21 days for six cycles [10-12]. Response to therapy is assessed with interim PET CT [6]. More aggressive regimens like R-DA-EPOCH are reserved for patients with high-risk diseases. Salvage Chemotherapy options with strategies like R-ICE or R-DHAP are considered in patients with refractory or relapsed diseases [11,12]. Following these, the eligible patients are considered for an Autologous stem cell transplant.

Given the aggressive nature and poor prognosis of relapsed/refractory THRLBCL, novel targeted therapies are gaining attention. CD19-directed CAR T-cell therapy, though not extensively studied in THRLBCL due to its rarity, has shown promising results in DLBCL and may offer benefits in select cases [13,14]. Similarly, venetoclax, a BCL-2 inhibitor, presents a potential therapeutic option, particularly in tumors expressing high levels of BCL-2 [15]. While evidence remains limited, both modalities represent rational approaches warranting further investigation in the management of refractory THRLBCL.

## Conclusions

T-cell/histiocyte-rich B-cell lymphoma represents a rare but aggressive variant of DLBCL that continues to challenge because of its varied presentations and striking histological overlap with reactive and infectious conditions. This case exemplifies how necrotizing and granulomatous lesions, particularly when accompanied by systemic symptoms, demand a diagnostic lens that extends beyond common infectious etiologies such as tuberculosis.

The case further highlights the critical role of adequately sampled biopsies coupled with an extended immunohistochemical panel in unraveling diagnostically elusive lymphomas. As the therapeutic landscape evolves with immunochemotherapy, targeted agents, and cellular therapies, early and precise diagnosis becomes indispensable to improve patient outcomes. Ultimately, by documenting such unusual presentations, we emphasize the necessity of cultivating broader awareness and diagnostic vigilance in order to avoid misclassification and ensure timely intervention in rare lymphoma subtypes.
